# *IJNS* Turns Seven—High Impact for Neonatal Screening

**DOI:** 10.3390/ijns7010016

**Published:** 2021-03-15

**Authors:** Ralph Fingerhut, Peter C. J. I. Schielen

**Affiliations:** 1 SYNLAB MVZ Weiden GmbH, Zur Kesselschmiede 4, 92637 Weiden, Germany; 2 Office of the International Society for Neonatal Screening, Reigerskamp 273, 3607 HP Maarssen, The Netherlands

Since our inaugural issue in 2015, the *International Journal of Neonatal Screening* (*IJNS*) has solidified its position as the preferred platform to publish the scientific output of the members of the International Society of Neonatal screening (ISNS) and professionals in fields related to neonatal screening. With the recent achievements of becoming a journal enlisted in PubMed Central (PMC), we have now achieved a place among scientific journals to be taken seriously. Below, we want to look back on the route *IJNS* has taken to get there, and our planned scheme for a road ahead.

*IJNS* can look back on six years of history. Founded in 2015, we could not have envisioned or foreseen whether our journal was going to be a success. Attempts in the past had not been fully successful, and so success was not guaranteed. For instance, would we be able to attract sufficient contributions for a viable scientific journal?

Secondly, we had to build up relationships with a new publisher, in a world that was relatively new to us. While open access has become one of the standards in scientific publishing, it was relatively new in 2013–2014. Would we be able to build up a readership, and become a constant in neonatal screening, if authors were to pay a fee for publication? Especially in a world where thousands of journals are available, and relatively few with an established reputation and high impact factor, many others were trying to find a niche in the increasingly competitive world of scientific publishing.

What would be the right format and how could we find and choose the right partner for publication? These were the questions we had in 2014, and most of the details were published in our first two editorials [1,2]. Our choice for a publisher was MDPI, and we are sure that we made the right choice.

For the first four years, we were able to waive all publication costs, which may have been encouraging for our first authors to send in their papers. We were able to advertise at various congresses, especially those of the ISNS and the Association of Public Health Laboratories (APHL), and that may have contributed to making a name for our journal.

Slowly but steadily, we were able to generate a steady number of contributions. Even within the first few years, we were able to reach some milestones. In December 2018, we became Scopus-enlisted, receiving our first CiteScore™ in May 2019, and also in May 2019, we became an ESCI (Web of Science)-enlisted journal.

At the end of 2017, 13 papers of *IJNS* were already listed in PubMed, mostly because these studies were funded by the NIH, or similar funding bodies, enabling PMC-enlistment. With the growing realization that we were establishing a sustainable journal, a second ambition was to be permanently and systematically enlisted in PubMed. In December 2017, we sent a first application to become PMC-enlisted to the National Library of Medicine (NLM).Here, the knowledge and guidance of our publisher, MDPI, should be mentioned prominently. Our colleagues at MDPI knew the proper channels for this application and guided the editorial board through the various procedures.

This first application failed, but the April 2018 review report of the NLM gave us insight into what was needed to improve. We have since then continuously worked to improve the quality of reviews (we now have a board of reviewers, and clear instructions were issued to focus on the specific points of the reviewer board), and we added three associated editors to our board.

A second attempt to obtain PMC indexing was more successful. In September 2020, *IJNS* passed the evaluation of the NLM for PMC indexing, which means all the papers published since 2018 in our journal will be indexed in PMC. As a result, if you type “Int J Neonatal Screen” into the search field of the PubMed website, you will find the 209 hits that comprise the 2018–2020 volumes, and 13 hits from former volumes (2015–2017).

Meanwhile, the number of submissions to *IJNS* has steadily increased, and so too has the number of published papers (Figure 1).

In addition, papers published in *IJNS* have been highly recognized, even before PMC indexing (Figure 2).

*IJNS* evolved into being the journal publishing the most papers in the field of neonatal screening, upholding that position in 2020.The council of ISNS and the managing editors of *IJNS*, together with the editors-in-chief and associate editors, have regularly discussed the policy steps of the journal. While in the first few years we tried to establish and build a core readership and authorship, and thus a sustainable journal, in the next few years we were encouraged to increase the yearly number of papers. We then started to invite Special Issue guest editors to start thematic Special Issues, gathering authors to publish their work on a given neonatal screening-related theme. Many of these Special Issues proved to be very successful, because of both the number of contributions as well as their quality, stimulated by the highly valued work of the guest editors. Due to the COVID-19 pandemic, the conference “SCID screening—State of the Art”, organized jointly by the ISNS and the UK Newborn Screening Laboratory Network, and originally planned for May 2020, was crafted into a two-day virtual meeting in January 2021. This meeting, a first experience of ISNS with virtual hosting, was quite a success, and *IJNS* and ISNS are now jointly working on a Special Issue, with the much-appreciated help of the presenters, as a spin-off of this meeting. Inspired by this first experience, the ISNS expects to organize similar single-issue conferences in the future, generating a cross-over between presentations and discussion in virtual meetings, and more elaborate reporting in *IJNS*.

After this, the management of the journal worked on expanding the editorial board and the board of reviewers. *IJNS* recruits high-quality reviewers that, for their valuable work, as a token of appreciation, receive a discount on the article publishing charge (APC) for publication in *IJNS*.

The success of *IJNS* also depended on the fact that regional associations for neonatal screening were prepared to affiliate with *IJNS*. Currently, the Association of Public Health Laboratories (APHL), ISNS, the German Society for Neonatal Screening (DGNS) and the Japanese Society for Neonatal Screening (JSNS) are affiliated with *IJNS* (these organizations stimulate their members to publish their work in *IJNS*, and *IJNS* offers support and often a considerable discount on the APC). We intend to invite more regional associations in the months and years ahead.

Now, we are at a new cross-roads of our journal. With the PMC enlistment, articles in *IJNS* are easier to find and read, and are thus obviously cited more frequently. Hopefully, Web of Science will grant *IJNS* its first Impact Factor in the near future as well. Since *IJNS* is the most prominent journal and platform for original papers in our field, and holds the most recent reports on many subjects, we expect that the best references for your upcoming papers can be found in *IJNS*.

ISNS is the licensee for *IJNS*, and with the growing success of the journal, new APCs were negotiated. The ISNS has tried to keep these as low as possible, and MDPI was generous enough to keep the APC at a modest price (lower than most benchmark journals). Current information on the APC can be found on the website of *IJNS* [3]. We hope that this low APC will continue to encourage our readership to choose *IJNS* as their preferred platform to publish original scientific results, opinions, technical reports, and everything in between. We also hope that with PubMed enlistment and possibly an Impact Factor, publication in *IJNS* will contribute to your H-index, and add to the value of the scientific output of your research groups.

We are very enthusiastic about the development of the journal. It serves to disseminate the high quality work of our members, the members of affiliated societies and, increasingly, the non-affiliated authors who find their way to *IJNS*. We hope that you, the readership of *IJNS*, will remain faithful to the cause and mission of our journal and of the society. We appreciate your support and will try to remain the best platform for the dissemination of all scientific work related to neonatal screening.

## Figures and Tables

**Figure 1:**
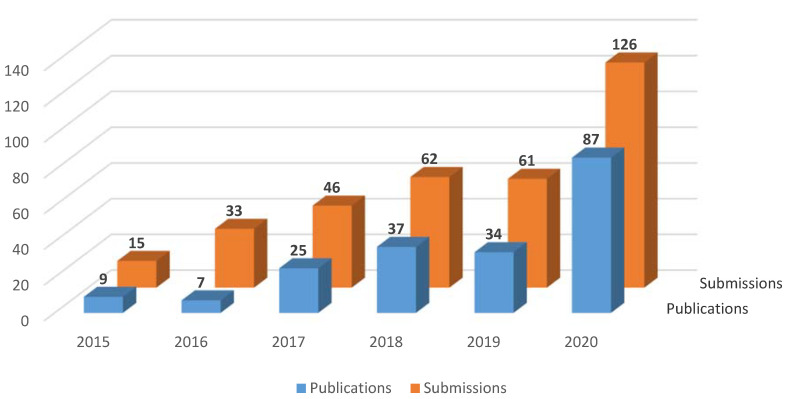
Number of submissions and publications (non-peer review article types such as editorials, corrections, obituaries, comments, replies, and meeting reports are excluded from the published papers) in 2015–2020.

**Figure 2:**
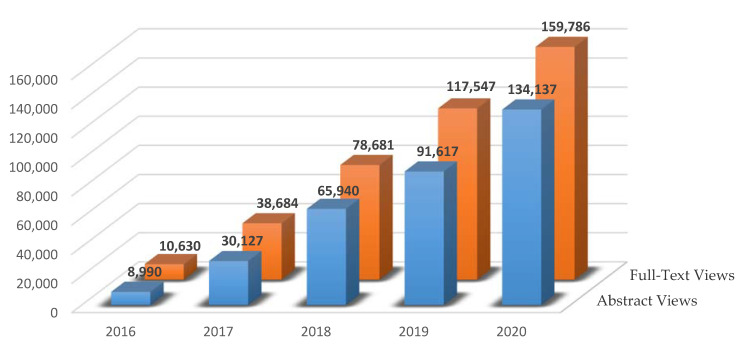
Total abstract and full-text views of *IJNS* publications in 2016–2020.
